# A Case Report of Subacute Infective Endocarditis Presenting With Extreme Weight Loss, Aortic Regurgitation, and Splenic Infarct

**DOI:** 10.7759/cureus.59866

**Published:** 2024-05-08

**Authors:** Austin Rahman, Patrick Rogers, Joshua B Piasecki, John Frederick

**Affiliations:** 1 Emergency Medicine, Lake Erie College of Osteopathic Medicine, Bradenton, USA; 2 Internal Medicine, Lake Erie College of Osteopathic Medicine, Bradenton, USA; 3 Internal Medicine, AdventHealth Florida, Tavares, USA

**Keywords:** strep viridans, aortic valve regurgitation, weight loss, subacute infective endocarditis, infective endocarditis

## Abstract

We present an insightful case of a middle-aged male who presented to the emergency department (ED) with complaints of excessive weight loss, accompanied by shortness of breath and vomiting. Consequently, this case explores many facets of the pathophysiology of infective endocarditis (IE), including but not limited to the most heavily implicated microorganisms, symptoms, predispositions, and disease outcomes. IE is a pathology of variable presentation with uniquely extensive diagnostic criteria, making it a fascinating topic of medical discussion.

## Introduction

Infective endocarditis (IE) is a prominent cardiac disease that is associated with a vast array of clinical presentations. It is often overlooked until hallmark symptoms (fever, murmurs, and embolic phenomena) become evident. Posing an even more significant challenge, IE can present insidiously with fewer debilitating symptoms (subacute), or with prominent and life-threatening symptoms within days (acute). Specific presentations can be described as classical features of IE, including splinter hemorrhages, Osler nodes, and Janeway lesions [1]. However, research has shown that these manifestations may not be as common as once suspected [1,2]. While the diagnosis of IE may be somewhat challenging, clinicians utilize criteria to aid them in the diagnosis: the Duke criteria. Consisting of microbiologic, histopathologic, clinical evidence, and imaging studies, most clinically diagnosed IE cases are done through the significant Duke criteria [3]. Regardless of the requirements mentioned above, the exact pathophysiology of IE is unchanged. IE is frequently associated with damaged cardiac valves, the majority of cases being the mitral valve, the aortic valve being the second most prominent, and tricuspid valve involvement usually being specific to intravenous (IV) drug users [4]. At its fundamental level, IE is the "seeding" of bacteria onto damaged valves, most commonly from gram-positive bacteria, although some gram-negative bacteria have been associated [4]. Although more common in underdeveloped populations, IE can often be misdiagnosed even in well-developed nations due to variable clinical manifestations. This case report looks into the intriguing findings of a patient with IE secondary to severe aortic regurgitation. 

## Case presentation

**Figure 1:**
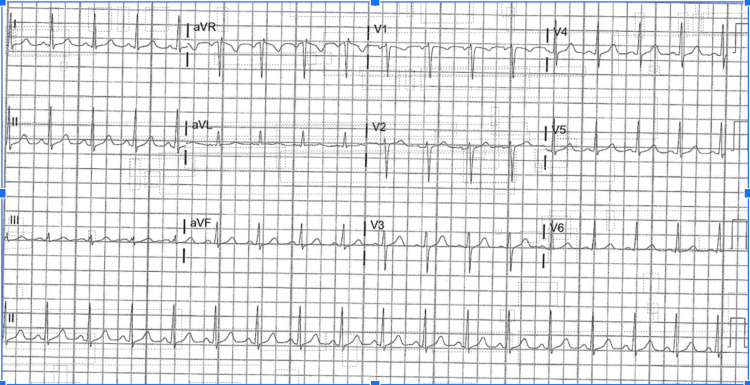
EKG-sinus tachycardia EKG: Electrocardiogram

Our 52-year-old only Spanish-speaking male patient presents to the hospital complaining of a two-month history of 40-lb weight loss, with occasional diarrhea. The patient states that he went to his primary care provider multiple times over the two months prior to referral to the emergency department (ED) for evaluation. The ED attending admitted our patient to the hospital floor based on the weight loss history, endorsement of shortness of breath with exertion, fatigue, and malaise. The patient also explained he has had recurrent nausea with nonbilious vomiting. He states that his stools are loose and his urine is "brown." Social history reveals that he has never smoked, consumed alcohol, or done recreational drugs. He is a truck driver by occupation and is married with two adult children. The patient's medical history is only positive for gout, for which he takes allopurinol; otherwise, he has no other ailments and has never undergone hospitalization or surgery. In the ED, the patient's vitals were a temperature of 36.9°C, heart rate of 100, respiratory rate of 18, blood pressure of 158/48 mmHg, and oxygen saturation of 98%. The patient's initial physical exam was normal, with no notable gross lesions, excoriations, or hemorrhaging present; cardiac, pulmonary, abdominal, and musculoskeletal exams were unremarkable in the ED. Basic workups in the ED included a complete blood count, a comprehensive metabolic exam, proBNP, a urine analysis (Table 1), and an electrocardiogram (EKG) (Figure 1) (which showed sinus tachycardia, otherwise normal). The patient's initial lab findings showed anemia at 9.3 hemoglobin, with a mean corpuscular volume of 88.6 fL. ProBNP was elevated at 949.8.

**Table 1:** Lab values WBC: white blood cell; RBC: red blood cell; MCV: mean corpuscular volume; MCH: mean corpuscular hemoglobin; MCHC: mean corpuscular hemoglobin concentration; RDW: red cell distribution width; MVP: mean platelet volume; CMP: comprehensive metabolic panel; BUN: blood urea nitrogen; proBNP: pro B-type natriuretic peptide; AST: aspartate transferase; ALT: alanine transaminase

CBC	Reference	Admission
WBC	4.40-10.50 10*3/uL	8.8
RBC	4.00-5.5.65 10*6/uL	3.27
Hemoglobin	12.6-16.7 g/dL	9.2
Hematocrit	36.9-48.5%	29.6
MCV	82.4-99.3 fL	90.5
MCH	27.5-34.1 pg	28.1
MCHC	31.7-36.1 g/dL	31.1
RDW	11.4-14.9%	12.5
Platelet count	139-361 10*3/uL	216
MPV	9.7-12.5 fL	10.9
Neutrophil %	50-70%	77.9
Lymphocyte %	1-15%	14.7
Monocyte %	1-15%	6.6
CMP		
Sodium	135-145 mmol/L	131
Potassium	3.5-5 mmol/L	4.5
Chloride	98-110 mmol/L	101
Carbon dioxide	24-31 mmol/L	19
Anion gap	5-15 mmol/L	-
BUN	5-25 mg/dL	19
Creatinine	0.6-1.20	1.29
Glucose	70-100 mg/dL	104
Calcium	8.5-10.5	8.4
AST	4-46 U/L	69
ALT	4-51 U/L	86
Alkaline phosphatase	40-129 U/L	46
Protein, total	6.5-8.0 g/dL	6.8
Albumin	3.20-5.50 g/dL	3.1
Bilirubin, total	0.10-1.5 mg/dL	.7
Biomarkers		
N-terminal ProBNP	0-125 pg/mL	949.8

**Figure 2:**
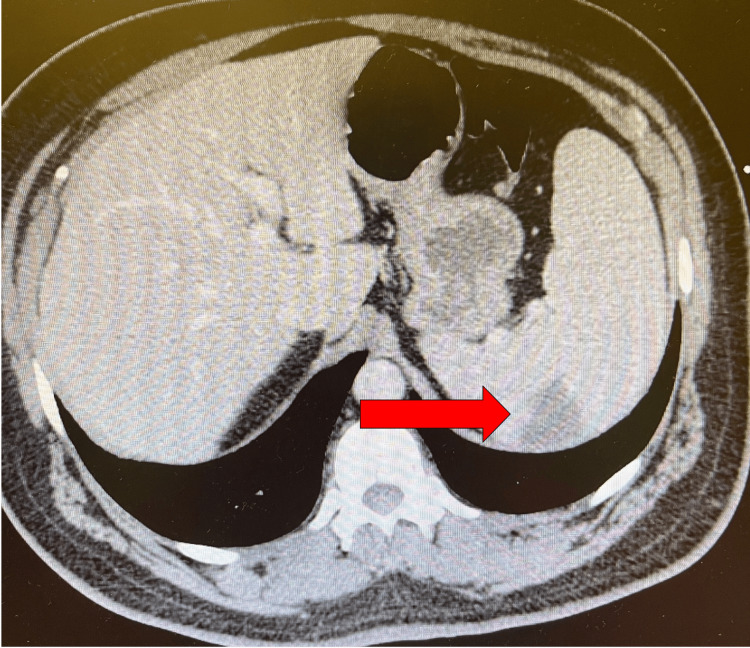
Splenic infarct seen on CT imaging

**Figure 3:**
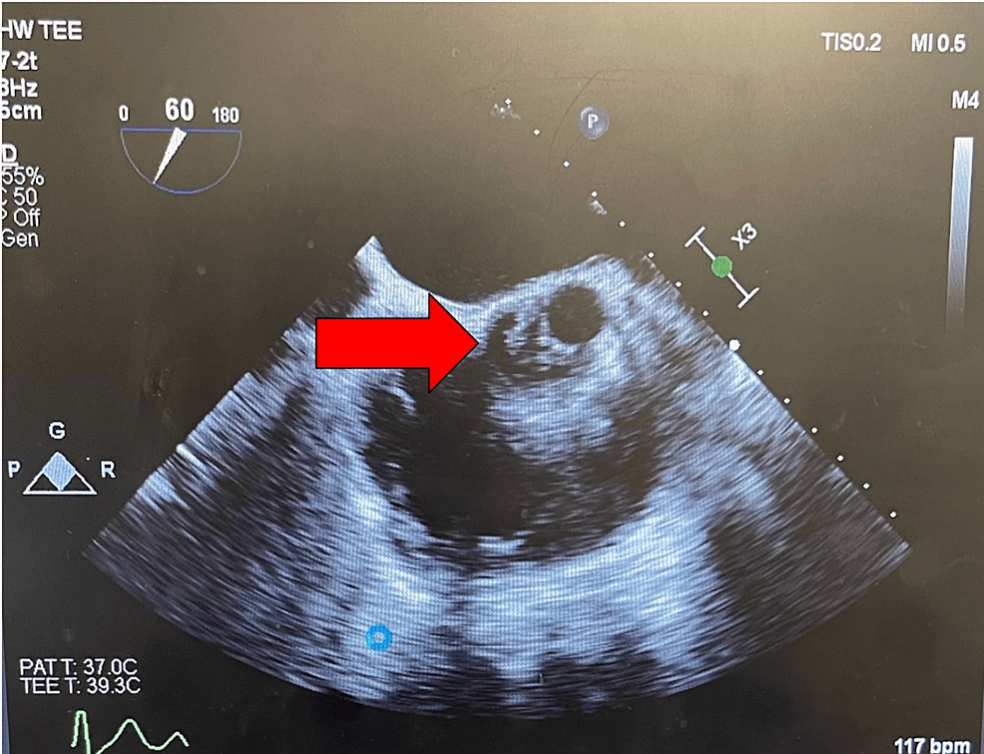
Extensive vegetation and aortic regurgitation shown on TEE TEE: transesophageal echocardiogram

Upon admission, a thorough physical exam revealed a "blowing" 4/6 diastolic murmur at the left sternal border and poor dentition; no skin findings or deformities were noted, and pulses were 2/2 in all four extremities. Further workup included a CT angiogram of the chest, a CT abdomen, and an X-ray of the chest. The findings of these imaging studies showed a splenic infarct (Figure 2) on the CT abdomen and pulmonary congestion on X-ray; otherwise, the imaging was benign. The splenic infarct was of particular concern, and a transthoracic echocardiogram (TTE) was ordered to rule out cardiac cause; cardiology was also consulted. Upon review of TTE, cardiology found a severe aortic regurgitation, and a follow-up transesophageal echocardiogram (TEE) was done. TEE confirmed the severity of the aortic regurgitation and revealed significant vegetation on the aortic leaflets (Figure 3). Left ventricular ejection fraction (LVEF) of 55% was appreciated. Cardiothoracic surgery was consulted, and infectious disease was consulted for analysis of suspected endocarditis; the patient was started on empiric IV vancomycin, 1.5 g. Blood cultures showed *Streptococcus viridans*, and the patient was switched to IV ceftriaxone 2 g. Based upon these findings and the likelihood of the source of infection being from oral flora, an oral maxillary surgeon was consulted for clearance to precede cardiothoracic surgery. Oral-maxillary consultation cleared the patient for cardiothoracic surgery. The patient was on IV antibiotics, and medical protocol dictated antibiotic management. The patient was eventually scheduled and successfully treated via an open-heart valve replacement of the said valve.

## Discussion

The patient in this case study is an example of subacute IE. The literature states that *Streptococcus viridans* is a common bacteria associated with poor dental hygiene [5]. The main problem was not the bacteria but that it was secondary to the severe aortic regurgitation. Figure 1 shows vast calcifications in the aortic valve; the vegetation is considerably large, so it can be inferred that the damage to the aortic valve happened over time. Literature states that vegetation of a specific size(s) indicates the mortality and associated embolic events that make IE lethal [6]. The patient's CT imaging showed a splenic infarct, which can be reasonably argued to be of embolic origin, secondary to endocarditis. Although the vegetation sizes were not measured, the TEE imaging shows the gross severity of the issue. Thus, the underlying proposed pathology behind our patient is that he had chronic aortic regurgitation, which was a faulty valve; this valve, over time, allowed vegetation to occur. The vegetation was then subsequently infected by *Streptococcus viridans*, likely from poor oral hygiene and dental caries.

Weight loss was the chief complaint that brought the patient into the hospital. Although weight loss is not generally associated with acute IE, it correlates with subacute endocarditis [7]. The patient described a 40-lb weight loss over a short period; this is considered a red flag in modern medicine, and as such, a proper workup should have been initiated to find the underlying cause. Our patient also complained of loose stools, nausea, and vomiting. All of these symptoms are classically seen with advanced malignancy of cancer, a very strong candidate for a differential [8].

It should be noted that a 40-lb weight loss within two months is considered extreme, even in the course of malignancy. If a CT abdomen had been done earlier, the splenic infarct could have prompted an earlier diagnosis. Given his bloodwork, the patient may have other underlying pathologies, but his IE is the most prominent and likely primary source of his clinical manifestations. As such, clinical practice dictates that it is the most urgent problem to be addressed. 

Duke's criteria is a primary diagnostic tool used to diagnose IE, and this case makes no exception to the rule. However, it should be noted that the three significant findings of Duke's criteria are the most clinically significant [3]. The three primary criteria are vegetation noted on imaging, positive blood cultures, and a new murmur [3]. A clinical diagnosis requires two major criteria [3]; our patient had all three. Heart sounds are a routine and staple clinical exam procedure clinicians do worldwide. Unfortunately, upon looking at the patient's prior clinical notes, no murmur was ever noted by any clinicians until arrival on the hospital floor; the patient was also unaware of the finding. Although this would not have changed the overall clinical outcome of the patient, as only two of the three criteria are needed for diagnosis, the patient may have been able to seek medical treatment earlier if the regurgitation murmur had been caught in prior encounters. Clinical literature states that diastolic murmurs, in this case, aortic regurgitation, are pathological and require follow-up by specialists [9,10]. 

The exact cause of why this patient had severe aortic regurgitation is an unanswered question. All consultants took our patient history thoroughly, and congruity was reflected in all notes. Aortic regurgitation has many etiologies, and thus, it would be difficult to pinpoint the exact cause of our patient's regurgitation [11]. It is important to understand that the aortic valve is not usually the most common valve affected in IE. As such, it is interesting to ponder why such extensive valvular vegetation was present in this patient [4]. The patient denied any form of prior medical history of infections, systemic disease, IV drug use, and past cardiac history. The patient with a new prosthetic heart valve will need to be treated prophylactically for specific procedures to prevent the recurrence of endocarditis [12]. It should be noted that the patient did not have a bicuspid valve, which often can lend itself to various pathologies, including IE [13].

## Conclusions

IE is a severe and often lethal manifestation of bacteria seeding associated with malfunctioning heart valves. Due to its extreme nature, clinicians must be vigilant and keep the endocarditis as a possible differential when patients present with vague symptoms. The authors believe clinicians must be alarmed by clinically pertinent, even subtle, murmur sounds. Murmurs are often overlooked, especially in present clinical care, where imaging and other diagnostic measures are paramount in clinical decision-making. The authors urge the medical community to be vigilant of subacute IE, its risk factors, and the importance of the physical exam. 

## References

[1] Hirai T, Koster M (2013). Osler's nodes, Janeway lesions and splinter haemorrhages. BMJ Case Rep.

[2] Servy A, Valeyrie-Allanore L, Alla F (2014). Prognostic value of skin manifestations of infective endocarditis. JAMA Dermatol.

[3] Topan A, Carstina D, Slavcovici A, Rancea R, Capalneanu R, Lupse M (2015). Assesment of the Duke criteria for the diagnosis of infective endocarditis after twenty-years. An analysis of 241 cases. Clujul Med.

[4] Yallowitz AW, Decker LC (2024). Infectious Endocarditis. https://www.ncbi.nlm.nih.gov/books/NBK557641/.

[5] Desimone DC, Tleyjeh IM, Correa de Sa DD (2012). Incidence of infective endocarditis caused by viridans group streptococci before and after publication of the 2007 American Heart Association's endocarditis prevention guidelines. Circulation.

[6] Berdejo J, Shibayama K, Harada K (2014). Evaluation of vegetation size and its relationship with embolism in infective endocarditis: a real-time 3-dimensional transesophageal echocardiography study. Circ Cardiovasc Imaging.

[7] Chong Y, Han SJ, Rhee YJ, Kang SK, Yu JH, Na MH (2016). Classic peripheral signs of subacute bacterial endocarditis. Korean J Thorac Cardiovasc Surg.

[8] Scheel BI, Holtedahl K (2015). Symptoms, signs, and tests: the general practitioner's comprehensive approach towards a cancer diagnosis. Scand J Prim Health Care.

[9] Dornbush S, Turnquest AE (2024). Physiology, Heart Sounds. https://www.ncbi.nlm.nih.gov/books/NBK541010/.

[10] Crawley IS (1990). Diastolic murmurs. Clinical Methods: The History, Physical, and Laboratory Examinations.

[11] Dewaswala N, Chait R (2024). Aortic Regurgitation. https://www.ncbi.nlm.nih.gov/books/NBK555944/.

[12] Thornhill MH, Dayer M, Lockhart PB, Prendergast B (2017). Antibiotic prophylaxis of infective endocarditis. Curr Infect Dis Rep.

[13] Kahveci G, Bayrak F, Pala S, Mutlu B (2009). Impact of bicuspid aortic valve on complications and death in infective endocarditis of native aortic valves. Tex Heart Inst J.

